# Insect responses to seasonal time constraints under global change are facilitated by warming and counteracted by invasive alien predators

**DOI:** 10.1038/s41598-024-76057-x

**Published:** 2024-10-19

**Authors:** Szymon Sniegula, Robby Stoks, Maria J. Golab

**Affiliations:** 1 Institute of Nature Conservation Polish Academy of Sciences, al. Adama Mickiewicza 33, 31-120 Kraków, Poland; 2 Evolutionary Stress Ecology and Ecotoxicology, University of Leuven, Leuven, Belgium

**Keywords:** Biological invasions, Carry over effects, Complex life cycle, Ecological interactions, Non-consumptive effects, Time constraints, Ecology, Evolution, Zoology, Ecology

## Abstract

In seasonal environments, organisms with complex life cycles not only contend with seasonal time constraints (TC) but also increasingly face global change stressors that may interfere with responses to TC. Here, we tested how warming and predator stress imposed during the egg and larval stages shaped life history and behavioural responses to TC in the temperate damselfly *Ischnura elegans*. Eggs from early and late clutches in the season were subjected to ambient and 4 °C warming temperature and the presence or absence of predator cues from perch and signal crayfish. After hatching, larvae were retained at the same thermal regime, and the predator treatment was continued or not up to emergence. The late eggs decreased their development time, especially under warming and when not exposed to predator cues. However, the late eggs increased their development time when exposed to predator cues, especially to crayfish cues. The TC decreased survival of late larvae that were as eggs exposed to crayfish cues, indicating a carry-over effect. The TC and warming additively reduced late larvae development time to emergence. Independent of the TC, predator cue effects on development time were stronger during the egg than during the larval stage. The late individuals expressed lower mass at emergence, which mirrored the size difference between field-collected mothers. Warming caused a higher mass at emergence. The late individuals increased their boldness and showed a higher number of moves, whereas warming caused a decreased boldness. There was no predator cue effect on larval behaviour. The results indicate that late individuals compensate for late season egg laying, which is facilitated under warming but counteracted under predation risk, especially when imposed by the crayfish.

## Introduction

In regions with temperate climates, the evolution of developmental traits in ectotherms may be influenced by time constraints (TC) that are driven by seasonal variation in temperature^1^. Changes in ectotherms’ developmental traits often affect body size and age at metamorphosis—traits closely related to fitness^2^. Theory and empirical evidence indicate a trade-off between both traits^3,4^. With some exceptions^5^, an early metamorphosis often comes with the cost of a smaller size^6^. This trade-off can be (partly) offset by an increased growth rate, enabling a short development to be combined with a large size at metamorphosis^7–9^. Yet, behavioural and physiological responses that underlie an increased growth rate may produce developmental and ecological costs, which may eventually decrease ectotherms’ juvenile and adult survival^1,10,11^. For instance, it was reported that increased larval activity rates in time-stressed insects lead to more effective food acquisition^12,13^, yet this comes with an increased predator encounter rate, leading to a trade-off between prey activity and predation risk^14,15^. As a result, measuring egg-to-adult developmental traits with associated costs is essential for unravelling the link between age and size at emergence under time stress.

Life history trade-offs related to seasonal TC can be further mediated by other ecological factors, such as warming and predation risk. Similarly as with time stress, warming is generally expected to increase ectotherms’ developmental parameters and decrease size at metamorphosis^16^. Predation risk may, however, alter these responses^2^. For instance, while warming decreased, predator cues increased larval development time until metamorphosis in a temperate damselfly^5^. As the effects of TC can be strongly modified and even reversed by warming and predation risk, it is important to evaluate responses to TCs under these widespread stressors.

Predators play a key role in shaping prey responses to TCs and warming^9,17^, but this may vary depending on predator species, which employ different hunting strategies, such as feeding on sedentary prey versus actively hunting moving prey^18,19^. For example, a number of crayfish species use sedentary aquatic organisms such as gastropods and bivalves as prey^20,21^, whereas predatory fish hunt actively moving invertebrates and vertebrates^22^. The hunting strategy may also affect which prey life stage is more vulnerable. For example, the egg stage is likely more vulnerable to predators feeding on sedentary prey as compared to predators feeding on moving prey. Overall, based on the prey sensitivity hypothesis^23^, the more dangerous a predator is, the more capable the prey should be at recognizing and responding to it.

The potential responses of organisms to ecological stressors may strongly depend on the developmental stage, especially when dealing with species showing a complex life cycle characterized by discrete egg, larval, and adult stages^24^. Furthermore, it is important to consider whether the effects of stressors experienced within a particular stage carry over to the subsequent stage. Such carry-over effects can have implications for overall fitness^25,26^. For example, stressors such as TC^27–29^, predation risk^30–33^ and warming^9^ may affect growth and development rates in the juvenile stage, resulting in delayed metamorphosis and/or a smaller adult size. In this context it is important to note that the egg stage is much less considered. Nevertheless, stressors imposed in the egg stage may affect the juvenile and even the adult stage. As an example, frog eggs that were exposed to predator cues underwent metamorphosis earlier and exhibited a larger body size^34^. Hence, to obtain a more complete understanding of the costs of responses to TC and how these are modulated by other stressors, a focus on multiple life stages is needed.

Here, we tested to what extent warming and predation risk shape the behavioural and life history responses to seasonal TC in an insect with a complex life cycle. Special attention is going to the differential impact of predation risk imposed by a crayfish versus a fish predator, feeding on sedentary benthic vs actively moving prey, respectively. We studied the blue-tailed damselfly *Ischnura elegans*, a semi-aquatic ectotherm which goes through aquatic egg and larval stages separated by metamorphosis from a terrestrial adult stage. We manipulated TC by comparing offspring from mothers caught early or late (less and more time constrained, respectively) in the breeding season. It has been shown that damselflies react to seasonal TC, predation risk and warming both by changing behaviour and life history^9,32,35–38^. We hypothesise that (1) late season eggs and the associated larvae will take shorter time for their development (time need to complete a life stage) than early season eggs and larvae, leading to a smaller size at metamorphosis, unless larval growth rate (mass accumulation within a life stage) is increased^7^. To accelerate life history, we also expect late larvae to show a higher activity and boldness^13,39^. (2) While warming is expected to increase the life history response to TC^40^, predation risk is expected to decrease the life history response to TC^41^. (3) Because of the different predator hunting strategies, we expected the effect of predation risk on immobile damselfly eggs to be stronger when imposed by a substrate-feeding crayfish predator and the effect of predation risk on mobile damselfly larvae to be stronger when imposed by the fish predator.

## Material and methods

### Study species

*Ischnura elegans* is a common Eurasian damselfly, neither protected nor endangered in Europe^42^. Adult females oviposit eggs into plants and debris at the water surface. Oviposition happens in late spring and summer, and hatching takes place a couple of weeks after eggs have been laid. Overwintering occurs as a diapausing larva. In central Europe, the species can complete either one or two generations within a year, i.e. shows a uni- or bivoltine life cycle, respectively, and variable voltinism has been reported within the same population^43–45^. Voltinism is determined by genetic and environmental factors^46^ and in seasonal environments might depend on hatching date within a growth season, thermal conditions and photoperiod experienced by larvae and other ecological factors such as predation risk^5,9,46,47^. Late-season egg laying and hatching impose TC on individuals to attain a threshold larval size (or instar) for successful overwintering (in univoltine cohorts) or to achieve emergence before the end of the same season in which the eggs were laid (in bivoltine cohorts)^48^.

Eggs and larvae commonly share water bodies with top predators such as fish and crayfish to which they respond with changes in life history traits^5,32,33,35,37^. Cues from two different predators were used to induce non-consumptive effects (NCEs). The first predator species was the native European perch (*Perca fluviatilis*) which is a common fish species in Europe^49^. The juveniles feed on moving prey such as zooplankton, invertebrates and other perch fry, whereas adults feed on moving invertebrates and fish^22,49^. Based on the perch feeding tactic, we expect that this fish poses greater danger to the active damselfly larvae than the inactive eggs. The study pond where eggs of *I. elegans* were collected contained perch. The second omnivorous but preferentially predator species was the invasive alien signal crayfish (*Pacifastacus leniusculus*) that has not co-occurred with *I. elegans* in the study pond. Yet, at least until 2015 the study pond contained another crayfish species, namely Danube crayfish (*Astacus leptodactylus*) (Bonk M., pers. comm.). Signal crayfish is native to North America. The crayfish was introduced to Europe, and Scandinavia specifically, in the 1960s. In the following years, the species was introduced in other European countries, including Poland (mainly northern regions)^50^, where it often co-occurs with *I. elegans*. *P. leniusculus* has a negative impact on local biodiversity. The crayfish has a significant potential to affect freshwater ecosystems, mainly through their consumption of invertebrates (preferentially slow moving or immobile prey) and plants. The crayfish tend to reach high population densities, exhibit greater consumption rates than native European crayfish, and colonize new areas more rapidly than native crayfish species, increasing their negative ecological effects^51,52^. The crayfish expressed faster growth rate when the substrate contained invertebrates^53^. Because the crayfish preferentially feeds on slow moving or immobile benthic prey, we expect that *P. leniusculus* poses greater danger to damselfly inactive eggs than active larvae. The nearest crayfish population is situated ca. 200 km from the damselfly study pond (Maciej Bonk and Rafał Maciaszek, pers. communication).

### Collection and housing of *I. elegans*

Part of the data analysed in this article was used in another article where *I. elegans* originating from different ponds (each pond with different predator species history and annual temperatures) were compared in terms of life history traits^5^. The focus of the previous study was not on TC, and behavioural traits were not examined.

Adult female *I. elegans* in copula were collected from Płaszowski pond in Kraków, Poland (50° 02′ 26.6″ N 19° 58′ 15.1″ E) on 5 July 2020 (hereafter, the early group; N = 14 females) and 8 August 2020 (hereafter, the late group; N = 12 females). After collection, females were placed in plastic containers (10 × 10 × 7 cm) with wet filter paper for egg laying. Plastic containers were placed in a Styrofoam box and transported by car to the Institute of Nature Conservation, Polish Academy of Sciences (INC PAS) where the laboratory experiment was carried out. Containers with females were kept in a room with a natural photoperiod and at 22 °C until females laid a single clutch of eggs, which took up to three days. After egg laying, adult females were preserved in 70% ethanol for size measurements. Experimental eggs and larvae were reared in four climate chambers.

### Experimental procedure

**Fig. 1:**
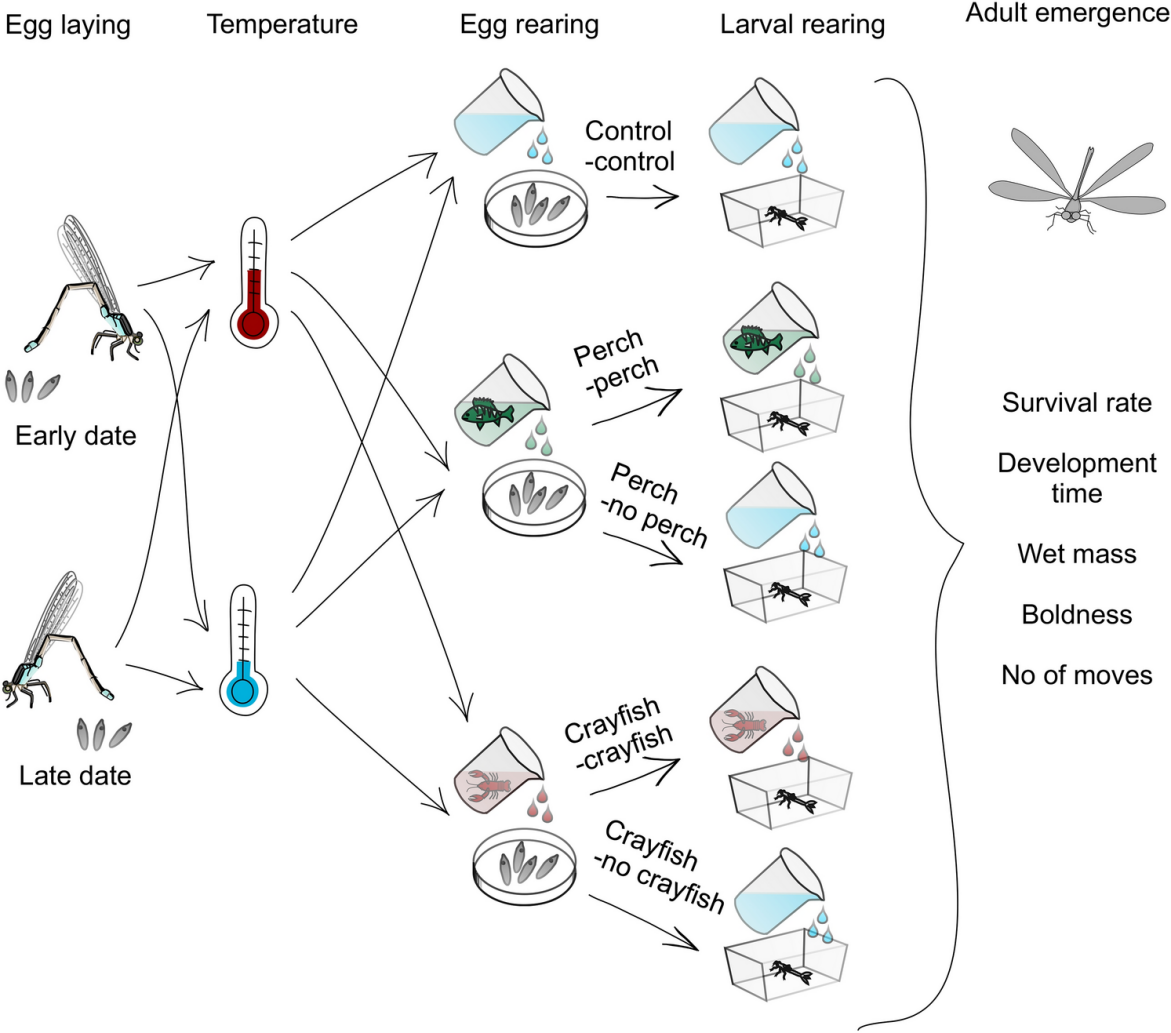
Graphical scheme of the experimental design testing for the effects of warming and predation risk on the response of eggs and larvae to TC associated with laying date. Early (less time constrained) and late (more time constrained) clutches of field-collected adult females were exposed to ambient and warming temperatures crossed with the three levels of predation risk: no predator cues, native perch cues and invasive alien signal crayfish cues. At hatching, control egg groups continued as control larval groups, whereas half of the perch and crayfish cue egg groups continued as perch and crayfish cue larval groups and half as control larval groups up to adult metamorphosis.

To test how warming and predation risk affect the responses to TC, we exposed early and late eggs to each of the six combinations of temperature (ambient or warming) and predator treatment (no cues, fish cues, crayfish cues) (Fig. 1). After hatching, larvae were kept at the same thermal regime as the eggs. Larvae that as eggs received the no cues treatment were maintained in the absence of predator cues in the larval stage; for larvae exposed in the egg stage to predator cues, one half were exposed to the same cues as the eggs while the other half were no longer exposed to predator cues. Groups that switched from predator cues to the control during the larval stage allowed evaluating carry-over effects of exposure to predator cues in the egg stage toward the larval stage.

Each egg clutch was divided into six sets and placed in separate plastic containers (15 × 11 × 7.5 cm) filled with 600 ml of water. Water consisted of ¾ dechlorinated tap water and ¼ of dechlorinated tap water with and without predator cues originating from European perch *P. fluviatilis* or signal crayfish *P. leniusculus*. Perch and crayfish cues were never combined, hence damselflies from different predator treatment groups experienced these predator cues separately. Eggs from each clutch were present in every treatment combination. At hatching, every predator treatment group during the egg stage was divided into two other groups: a group that continued experiencing predator cues during the larval stage and a control group that did not experience predator cues during the larval stage. Water with predator cues was partially changed every second day to keep the predator cue levels approximately constant, taking into account the time of cue biodegradation^54^. This time interval of two days has been shown to be sufficient to allow the predator cues to affect life history traits in damselfly eggs and larvae^36,38,55^.

Hatching took place a couple of weeks after eggs had been laid. Upon hatching, the larvae were moved into separate containers (19 × 12 × 9 cm) holding 1 l of water. Two of these containers were installed per initial egg treatment and each container contained 15 individuals (early group) and 20 individuals (late group), totalling 30 (early group) and 40 (late group) individuals per treatment at the start of the larval growth period. For the initial 14 days, larvae were kept in groups to increase larval survival^56^. After this time, every larva was individually placed in a plastic cup (height 9 cm, diameter 4 cm, volume 200 ml) filled with 100 ml of water. Water refills were analogous as in the egg stage (¾ of dechlorinated tap water and ¼ of dechlorinated tap water with and without predator cues; ¼ changed every second day). The larvae were fed twice a day (morning and afternoon) during weekdays and once a day during weekend days with 1 ml of laboratory cultured *Artemia* sp. nauplii (mean = 198.5, SD = 92.4 nauplii/1 ml, N = 38). During the first 14 days, larvae in groups received 10 ml of *Artemia* solution. Larvae kept in individual cups received 1 ml of solution. During the simulated winter (see description below) the larvae were fed once a day. When larvae reached the pre-final instar before emergence, they were additionally provided with one living chironomid larva three times a week (Monday, Wednesday and Friday). This supplementary food was given to each larva until adult emergence. The experiment ended one day after the last individuals emerged. The experimental design in a graphical format is presented in Fig. 1.

**Fig. 2:**
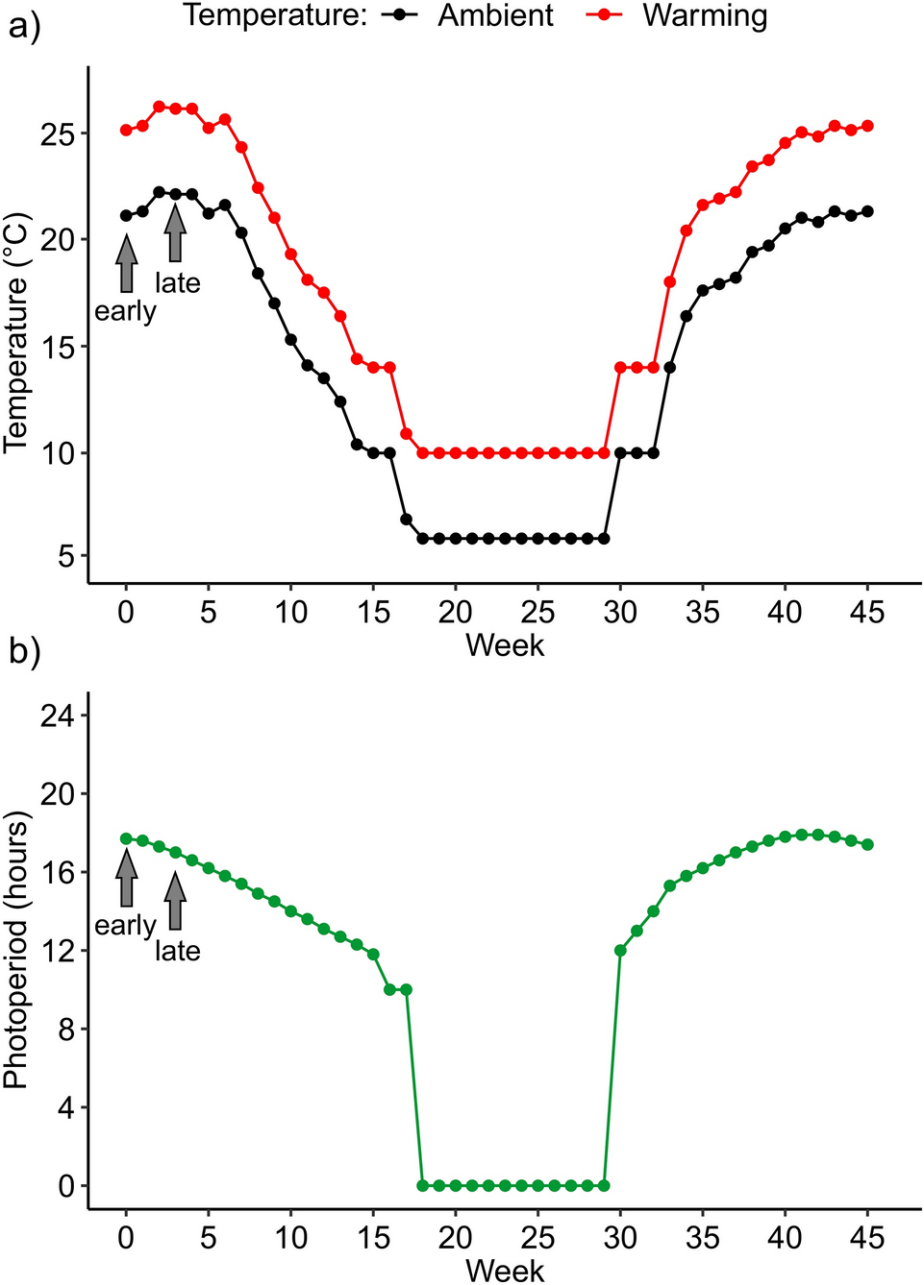
Temporal progress during the experiment of (**a**) temperatures (ambient and warming) modelled with the Flake function^58^, and (**b**) photoperiods. Each point matches a weakly change of the thermo-photoperiod. Arrows indicate early and late egg collection dates. Week 0 refers to 3 July. The simulated winter in weeks 18–29 matches 6 November–2 April real dates.

The temperatures at which eggs and larvae were reared were adjusted once a week to follow seasonal changes of mean weekly temperatures in the shallow part of ponds which are optimal for damselfly larvae^48^. The experiment consisted of two temperature treatments: the ambient temperature to mimic the ambient thermal conditions in the collection pond, and a + 4 °C higher temperature to mimic the predicted temperature change by 2100 under IPCC^57^ scenario RCP8.5 (Fig. 2a). The ambient temperatures were modelled with FLake model^58^. The simulated water temperature was estimated over the period 1998–2009 and included the following meteorological and pond-specific parameters: wind components, air temperature, humidity, cloud cover, solar and thermal radiations, geographic location (= coordinates), pond depth and water transparency. We followed weekly changes in day light (photoperiod) according to civil twilight length in Kraków (Fig. 2b). Because in nature *I. elegans* overwinters in the larval stage (second generation of the bivoltine cohort or first generation of the univoltine cohort), and so larvae experience a winter diapause we simulated winter conditions. During winter, we set a constant temperature of 6 °C (ambient temperature treatment) and 10 °C (warming temperature treatment). The light during winter was switched off. We acknowledge that the simulated winter in our laboratory experiment was significantly shorter than the actual winter period experienced in nature (Supplementary Fig. S1, Supplementary Table S1). During winter conditions, coenagrionid larvae continue feeding^59^, and as a result have been shown to increase in mass (for the study species^60^). The shorter winter duration in our lab experiment could potentially result in larvae showing less mass gain during winter compared to their counterparts in nature. However, it is important to note that during the wintering period, any mass increases are small, likely because of the reduced metabolic rates^46^.

To compare modelled water temperatures to the actual temperatures that *I. elegans* eggs and larvae experience in the study pond, we installed a datalogger at a depth of 40 cm (Hobo^®^ Pendant^®^ Data Logger UA-001-64). The datalogger recorded water temperatures four times per day between February and November 2023. These data revealed that during the first growth season, i.e., before the simulated winter, 3 July–30 October (experimental weeks 0–16) the actual field temperatures overlapped more with the experimental warming treatment than with the experimental ambient temperature treatment. The opposite pattern was found during the second growth season, i.e. after simulated wintering, 2 April–16 July (experimental weeks 30–45, including the simulated winter weeks). Plots and numerical tables with the mentioned temperatures are in Supplementary Figs. S1, S2 and Supplementary Table S1.

### Collection and housing of the predators

For a detailed description of collection and housing of the predators see Supplementary Methods. Briefly, perch and crayfish were collected a couple of weeks before we started the experiment. Perch was collected from Dobczyce Lake in southern Poland and signal crayfish from Hańcza Lake in northern Poland. Animals were transported to the INC PAS, separated by species and kept in aquaria with 52 l of tap water at a constant temperature of 20 °C. The densities of predators in aquaria were based on the basal metabolic rate equations^61,62^. Predators were fed with unfrozen chironomid larvae every second day and live earthworms once a week. Perch were collected and housed with permission from the Local Ethical Committee (ref. 394/2020). Signal crayfish were collected with permission from the Regional Directorate for Environmental Protection in Białystok (ref. WPN.6205.21.2020.ML) and Nature Reserve Hańcza Lake and housed with permission from the Regional Directorate for Environmental Protection in Kraków (ref. OP-I.672.8.2020.MK1). The collection and housing procedures were performed in accordance with relevant guidelines and regulations.

### Response variables

#### Life history traits

Early and late field-collected adult females whose clutches were used were photographed with a camera linked to a binocular microscope (SMZ 745T Nikon and camera DFK 23UP031 Nikon, Tokyo, Japan). Thereafter, head width was measured as measure of body size^48^. For the laboratory reared damselflies, individual survival was measured as the number of damselflies that survived during the first 14 larval days and until 1 day after emergence. Development time was measured as number of days between egg laying and egg hatching (egg development time), between hatching and adult emergence (larval development time), and between egg laying and adult emergence (total development time). We also quantified development times in degree days (DDs), i.e. summation of daily temperatures over a period of time in days, formulated as DD_summation_ = ∑ daily temperature, since DDs better capture the temperature-dependent nature of insect development^63^. We summed the daily temperature degrees experienced during the egg or larval stages. We applied a lower temperature threshold of 10 °C, excluding the winter period when calculating DDs in both temperature treatment groups. This threshold was chosen because the minimum temperature for larval development in Odonata typically falls within the range of 8–12 °C^64^. Adult wet mass was measured one day after emergence when the cuticle was hardened with an electronic balance to the nearest 0.01 mg (Radwag^®^ AS.62. R2 Plus).

#### Behavioural traits

All larvae were scored for two behavioural traits. Between one to three days after larvae entered the last instar prior to emergence, when their exoskeletons had hardened, behavioural observations started. To standardize hunger levels, larvae were not fed the day before the behavioural observations. Larvae were individually transferred from cups to larger containers (12 × 8 × 5 cm high) filled with water with or without predator cues depending on the predator treatment of the larvae. These containers were placed in room with an air temperature of either 24 °C (ambient temperature) or 28 °C (warming treatment). Before the observations began, larvae were given 30 min to acclimate to the containers.

Behavioural trials were initiated by recording the number of moves each larva performed based on a 2 × 2 cm grid drawn on the bottom of the containers. Movements were scored when separated by at least 2 s of larval immobility. Each movement trial lasted for 5 min. Immediately after the movement trial, we measured larval boldness. Within the same containers, each larva was gently poked at the caudal lamellae with a thin wooden stick, triggering a larval escape response followed by freezing (inactivity). The latency of the larva to start moving following freezing was used as a proxy for boldness, with a shorter freeze period indicating bolder larvae (as done for the study species in Tüzün et al.^39^. The maximum latency time was set at 5 min. If the larva did not move within this time, it was given a 5 min latency period.

During the course of the experiment, besides natural mortality we accidentally lost 27 individuals (Supplementary Table S2). Hence, some treatment groups ended up with low sample sizes and caution should be taken when interpreting the results.

### Statistical analyses

For statistical analysis we used R version 4.3.2^65^ The size of early and late field-collected adult females was compared with a t-test. The difference in survival rate between experimental groups was estimated with a generalized model (glm function) with a binomial error distribution. Egg, larval and total development times in days and degree days, wet mass, boldness and number of moves were analysed using separate linear models (lm function). Collection date (early and late date), temperature (current and warming), predator cue treatments and their interactions were explanatory variables. Note that for the egg stage there were three levels of the predator cue treatment: control, perch and crayfish cues, while in the larval stage there were five levels: control (egg)–control (larva), perch (egg)–control (larva), perch (egg)–perch (larva), signal crayfish (egg)–control (larva), and signal crayfish (egg)–signal crayfish (larva). In addition, in models analysing larval and total development time, mass, boldness and number of moves we also included sex (sex could not be determined during the egg stage and in 14-days old larvae). Analyses of boldness and number of moves were corrected by mass at emergence. Initially, we ran global models that included all main effects and interaction terms. Subsequently, the interaction terms with p > 0.1 were removed from the final models.

## Results

Sample sizes per combination of collection date, temperature, and predator cue treatment ranged between 14 and 79 eggs (median N = 38) and between 3 and 22 larvae (median N = 8). There was one group (early collection control-control group under ambient temperature) with a sample size of one (for survival until emergence, larval development time, and mass at emergence) or zero (for larval boldness and number of moves) individuals. After removing this group from the models analysing survival until emergence, larval development time and mass at emergence, the final results did not change qualitatively. Detailed numerical information with sample sizes per treatment group is provided in Supplementary Table S3.

### Field-collected adult females

Early collected females (mean head width 3.88 mm, N = 14) were 3% larger than late collected ones (mean head width 3.76 mm, N = 12) (t = 3.089, df = 24, p-value = 0.005).

### Egg development time

**Fig. 3:**
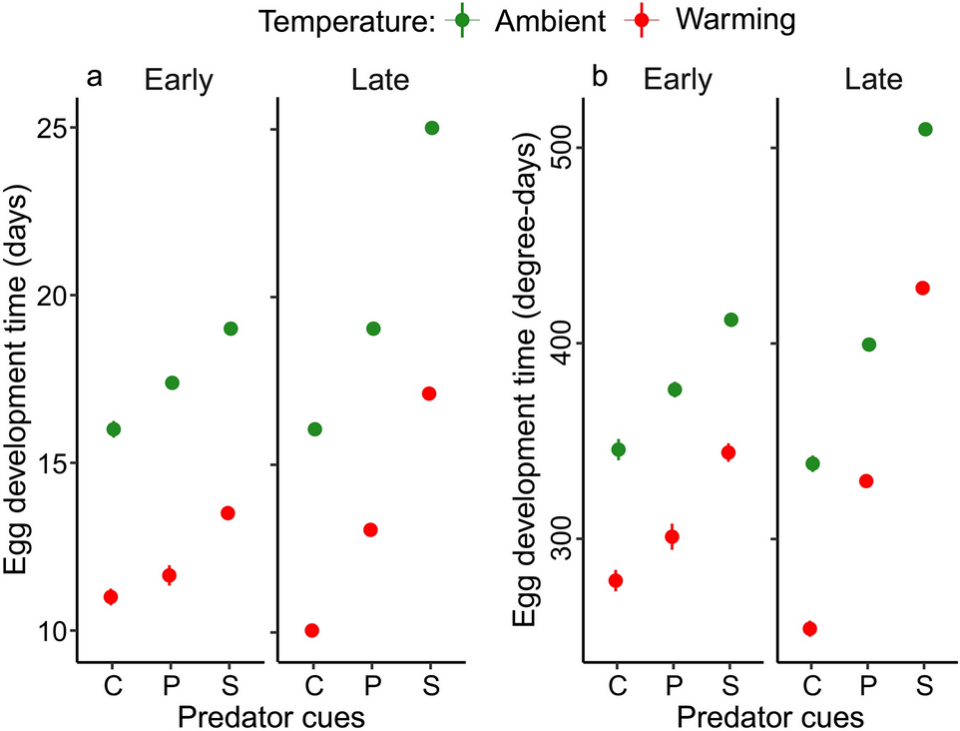
Estimated mean egg development time of *I. elegans* in days (**a**) and in degree days (**b**) between different collection dates (early and late), temperatures (warming and ambient) and predator cue treatments. Error bars show 95% CI. *C* control, *P* perch cue, *S* signal crayfish cue.

Overall, late individuals had a longer egg development time in days, and while warming reduced egg development time, predator cues increased it. Warming and predator cues interacted in shaping egg development time, and this interaction differed between early and late individuals (interaction collection date × temperature × predator cue) (Fig. 3a, Table 1). Warming shortened the egg development time especially in the late control group resulting in the shortest development time in days in this combination. Predator cues increased development time most strongly in the late group exposed to signal crayfish cues at the ambient temperature, resulting in the longest egg development time in days.

**Table 1:** Results from linear models testing for effects of collection date, temperature and predator cue on egg development time in days and degree-days.

Predictor	Df	F	P
Egg development time in days			
Collection date	1	2087.633	< 0.001
Temperature	1	14,832.491	< 0.001
Predator cue	2	4729.525	< 0.001
Collection date × temperature	1	157.860	< 0.001
Collection date × predator cue	2	820.183	< 0.001
Temperature × predator cue	2	82.749	< 0.001
Collection date × temperature × predator cue	2	34.692	< 0.001
Egg development time in degree-days			
Collection date	1	1324.537	< 0.001
Temperature	1	4266.816	< 0.001
Predator cue	2	4674.516	< 0.001
Collection date × temperature	1	12.621	< 0.001
Collection date × predator cue	2	667.916	< 0.001
Temperature × predator cue	2	3.835	0.022
Collection date × temperature × predator cue	2	7.028	< 0.001

Generally, the pattern of egg development time in degree-days (DD) aligned qualitatively with that of development time in days across different hatching dates and treatments (Fig. 3b, Table 1).

### Survival

**Fig. 4:**
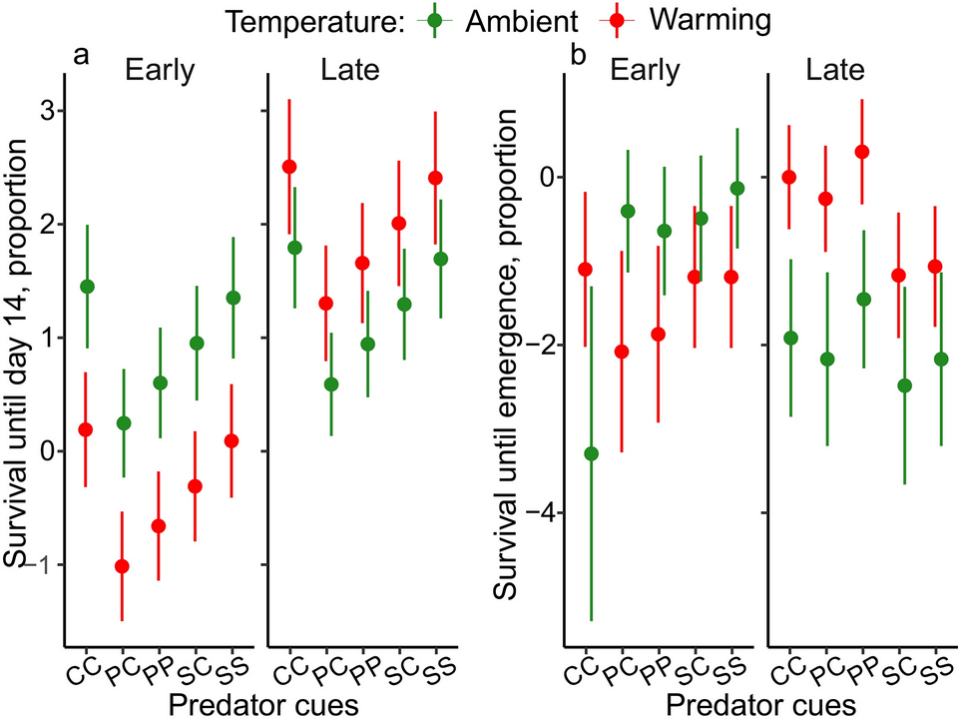
Estimated proportion of larval survival (**a**) during the first 14 days after egg hatching, and (**b**) until adult emergence for the different combinations of collection date, temperature and predator cue treatments. Error bars show 95% CI. *CC* control egg-control larva, *PC* perch egg-control larva, *PP* perch egg-perch larva, *SC* signal crayfish egg-control larva, *SS* signal crayfish egg-signal crayfish larva.

Late individuals had on average a higher survival during the first 14 days after egg hatching than early ones. Higher survival in late individuals and lower survival in early individuals were more pronounced under warming (interaction collection date × temperature). Exposure to predator cues negatively affected survival until day 14, but did not interact with other factors (Fig. 4a, Table 2).

**Table 2:** Results from linear models testing for effects of collection date, temperature, predator cue and sex on larval survival, development time in days and degree-days, and mass at emergence.

Predictor	Df	χ^2^	P
Survival to day 14			
Collection date	1	57.112	** < 0.001**
Temperature	1	4.140	**0.042**
Predator cue	4	25.139	** < 0.001**
Collection date × temperature	1	29.551	** < 0.001**
Survival to emergence			
Collection date	1	0.000	0.999
Temperature	1	9.452	**0.002**
Predator cue	4	4.13	0.388
Collection date × temperature	1	41.456	** < 0.001**
Collection date × predator cue	4	20.492	** < 0.001**
Collection date × temperature × predator cue	8	14.477	0.07

Predictor	Df	F	P
Larval development time in days			
Collection date	1	47.87	** < 0.001**
Temperature	1	310.332	** < 0.001**
Predator cue	4	1.747	0.141
Sex	1	7.115	**0.008**
Larval development time in degree-days			
Collection date	1	83.003	** < 0.001**
Temperature	1	41.375	** < 0.001**
Predator cue	4	1.665	0.16
Sex	1	8.066	**0.005**
Collection date × temperature	1	12.863	** < 0.001**
Total development time in days			
Collection date	1	29.422	** < 0.001**
Temperature	1	465.218	** < 0.001**
Predator cue	4	3.179	**0.015**
Sex	1	7.425	**0.007**
Collection date × temperature	1	6.147	**0.014**
Total development time in degree-days			
Collection date	1	59.791	** < 0.001**
Temperature	1	18.448	** < 0.001**
Predator cue	4	3.113	**0.017**
Sex	1	7.317	**0.007**
Collection date × temperature	1	14.365	** < 0.001**
Mass			
Collection date	1	54.506	** < 0.001**
Temperature	1	20.156	** < 0.001**
Predator cue	4	1.156	0.332
Sex	1	6.35	**0.013**
Collection date × temperature	1	25.048	** < 0.001**

Significant values are in bold.

Late individuals had a lower survival rate until emergence than early individuals, but only at the ambient temperature; the opposite pattern was found under warming (interaction collection date × temperature) (Fig. 4a, Table 2). Early and late individuals differently responded to warming and predator cues. At the ambient temperature, early damselflies showed decreased survival in the egg control-larval control than in the other predator cue groups. Under warming, late damselflies showed lower survival in the signal crayfish egg-control larva and signal crayfish egg-signal crayfish larva groups than in the other predator cue groups [interaction collection date × predator cue, and a trend for interaction collection date × temperature × predator cue (p = 0.07)] (Fig. 4b, Table 2).

### Larval development time

**Fig. 5:**
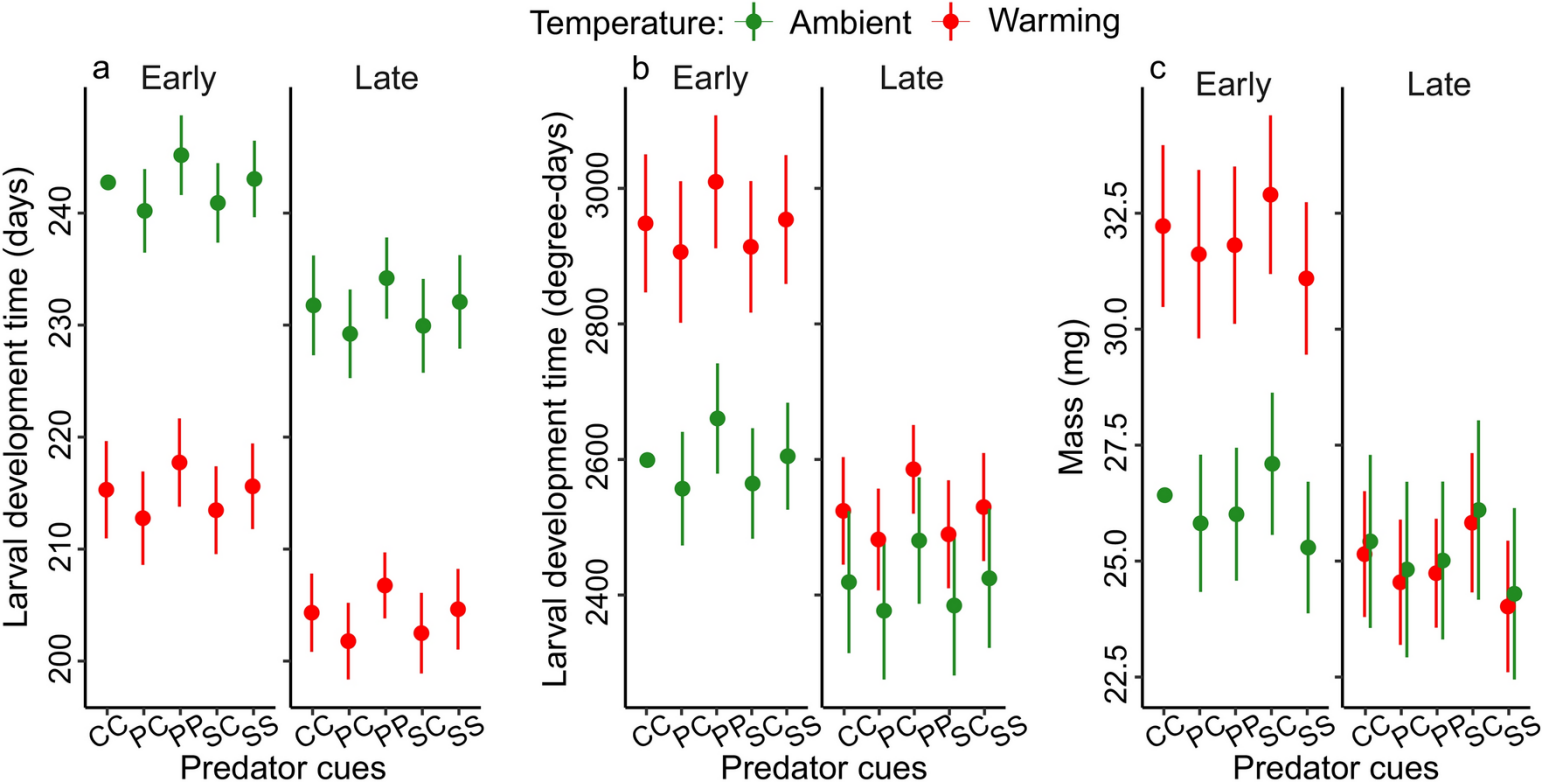
Estimated mean larval development time (**a**) in days and (**b**) in degree-days, and (**c**) mass at emergence for the different combinations of collection dates, temperature and predator cue treatments. Error bars show 95% CI. The sample size per group is provided in (**a**). *CC* control egg-control larva, *PC* perch egg-control larva, *PP* perch egg-perch larva, *SC* signal crayfish egg-control larva, *SS* signal crayfish egg-signal crayfish larva.

Late larvae had shorter development times (expressed in days) than early larvae. Warming shortened development time. Predator cues did not affect larval development time (Fig. 5a, Table 2). Females had a longer larval development time than males (Supplementary Fig. S3a, Table 2). No interaction terms were significant (Table 2).

Late hatchers required fewer degree-days (DD) than early hatchers, especially under warming. Late larvae at the ambient temperature needed the least DD, whereas early larvae under warming needed the most DD (interaction collection date × temperature). Predator cues did not affect larval DD development (Fig. 5b, Table 2). Females required more DD than males for larval development (Supplementary Fig. S3b, Table 2).

### Total development time

Total development time (in days) from egg hatching until adult metamorphosis was shorter in late individuals. Warming further decreased total development time, especially in late individuals (interaction collection date × temperature). Predator cues increased total development time (Table 2), which was pronounced in perch egg-perch larva, signal crayfish egg-control larva and signal crayfish egg-signal crayfish larva groups (Supplementary Fig. S4a). Females took longer time for total development time than males (Supplementary Fig. S5a, Table 2).

Similarly, late damselflies required fewer DD for total development time than early ones. Warming did not affect DD in late individuals, but increased DD in early individuals (interaction collection date × temperature). Predator cues increased DD (Table 2), which was pronounced in perch egg-parch larva, signal crayfish egg-control larva and signal crayfish egg-signal crayfish larva groups (Supplementary Fig. S4b). Females took longer time for total development in DD than males (Supplementary Fig. S5b, Table 2).

### Mass at emergence

Early individuals reached a higher mass at metamorphosis than late ones, but only under warming. Warming increased body mass in early, but not in late individuals (interaction collection date × temperature). Predator cues did not affect mass (Fig. 5c, Table 2). Females were heavier than males (Supplementary Fig. S6, Table 2).

### Boldness

**Fig. 6:**
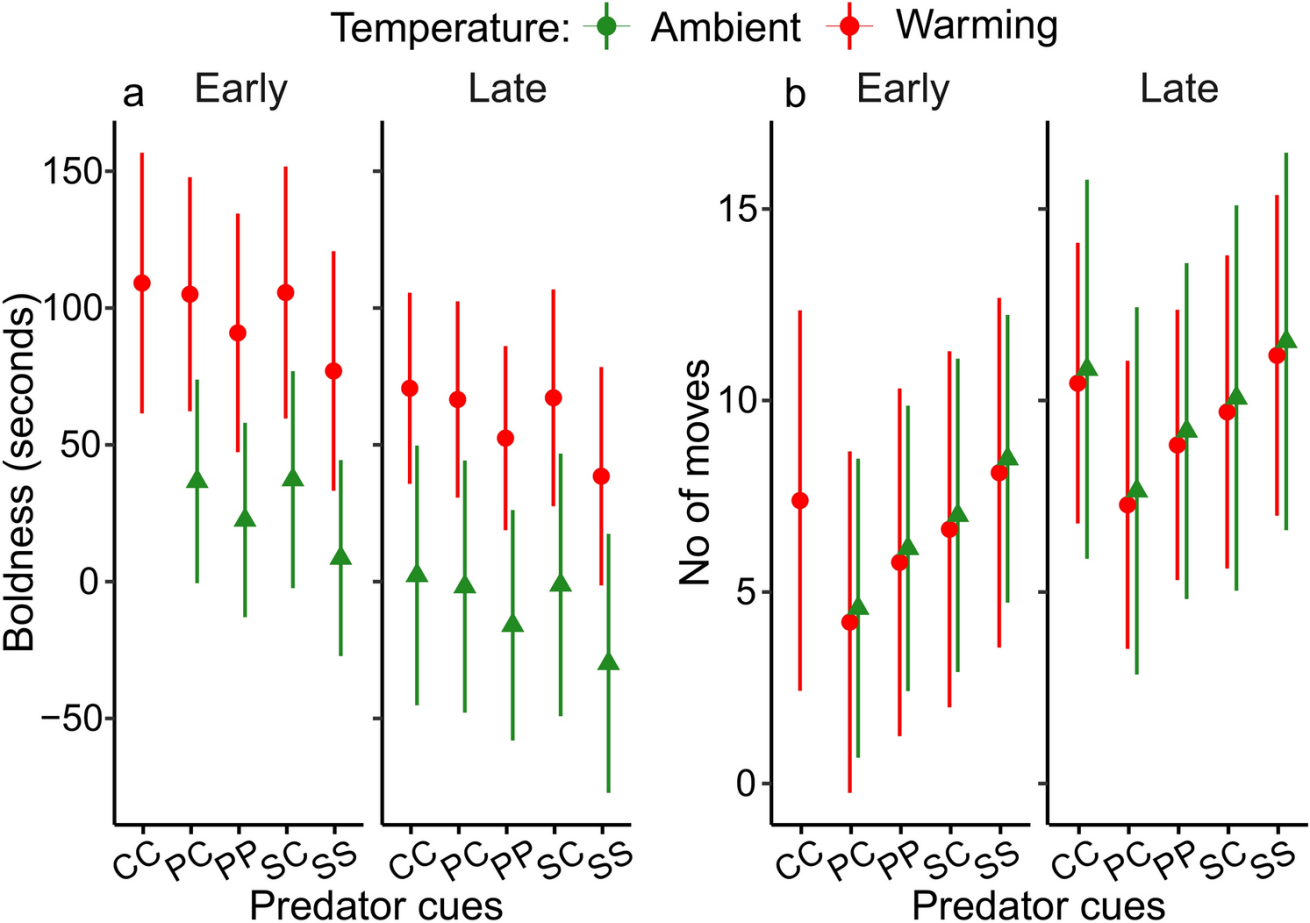
Estimated mean larval boldness (**a**) and number of moves (**b**) for the different combinations of collection dates, temperature and predator cue treatments. Error bars show 95% CI. The sample size per group is provided in (**a**). *CC* control egg-control larva, *PC* perch egg-control larva, *PP* perch egg-perch larva, *SC* signal crayfish egg-control larva, *SS* signal crayfish egg-signal crayfish larva.

Late individuals showed a higher boldness compared to early ones. Larvae were bolder when reared under the ambient rather than warming temperatures. Neither predator cues nor sex had a significant effect on boldness, and none of the interactions were significant (Fig. 6a, Table 3).

**Table 3:** Results from linear models testing for effects of collection date, temperature, predator cue and sex on larval boldness and number of moves. Boldness and number of moves were corrected by larval mass at emergence.

Predictor	Df	F	P
Boldness			
Collection date	1	4.567	**0.035**
Temperature	1	14.582	** < 0.001**
Predator cue	4	0.595	0.667
Sex	1	0.113	0.738
Mass	1	0.668	0.415
Number of moves			
Collection date	1	2.611	0.109
Temperature	1	0.030	0.862
Predator cue	4	0.677	0.609
Sex	1	0.461	0.499
Mass	1	0.027	0.869

Significant values are in bold.

### Number of moves

There were no main or interaction effects for any variable on the number of larval moves (Fig. 6b, Table 3). However, after excluding all explanatory variables except for the collection date from the model, the results for this variable became significant (p = 0.034): late individuals were more active than early ones.

## Discussion

We predicted a response to time constraints (TC) imposed by late egg hatching in life history and behavioural traits, and this response to be stronger under warming compared to the ambient temperature, and to be stronger in the absence of predator cues. Our results partially supported these predictions. While we found the predicted partial compensation for a late-season oviposition date in egg and larval development times, and the associated higher larval boldness and activity rate, compensation in development time was only pronounced under warming and in the absence of predator cues. Shorter development times resulted in a lower mass at emergence, an expected fitness cost for the accelerated life history under TC, yet no cost in terms of increased mortality rate was detected. As expected, predator cues delayed development time and, surprisingly, the negative carry-over effect of predator cues experienced in the egg stage was more pronounced in time-constrained damselflies, further delaying life history transition dates. Interestingly, cues from a generalist predator (signal crayfish), capable of preying on both aquatic stages of the studied prey (eggs and larvae), had a stronger effect on development than those from the more specialized predator (perch), which primarily hunts the mobile larval stage. This stronger crayfish effect was evident only during the egg stage, whereas both crayfish and perch cues similarly delayed total development time and did not affect mass at emergence.

### Seasonal compensation under warming and predator cues in the egg stage

Our results demonstrate partial compensation for late-season oviposition dates in egg development time, particularly under warming conditions and in the absence of predator cues. Faster egg development under time-stress situations has been reported in a few other temperate ectotherms^66–68^. For example, regardless of temperature conditions, eggs laid during the late season generally had shorter incubation periods compared to those laid early in the season in the lizard *Anolis sagrei*^68^. We report that such compensation also occurs in species with variable voltinism, where time stress can be alleviated by the addition of an extra growth season for juvenile development. This response appears to be adaptive, as earlier egg hatching might provide more time for larval development and growth before wintering, likely increasing winter survival and may reduce larval mortality caused by cannibalism, specifically between individuals from the same hatching date cohort^9,69,70^.

The observed slight temperature differences during the first two (warming treatment) and three-four weeks (ambient temperature treatment) of egg rearing experienced by early and late eggs are unlikely to have triggered the difference in egg development rate (Supplementary Table S1). In fact, during the first two weeks, the temperature was 0.2 °C higher in the late group. However, during the third and fourth weeks, the temperatures in the early and late groups reversed, with the early group experiencing lower temperatures by 0.5 °C and 1.3 °C, respectively, further supporting weak or no temperature-caused difference in hatching dates between early and late groups. However, the results based on thermal units, degree-days (DDs), differed from these based on development time in days. Specifically, a larger difference in hatching dates between early and late groups was observed when egg development time was expressed in DDs (compare Fig. 3a vs. Fig. 3b). The somewhat shorter photoperiod encountered by late eggs could be a contributing factor. Seasonally changing day length indicates the remaining time window available before the end of the growth season. Previous studies have highlighted the important role of seasonal changes in photoperiod in shaping hatching phenology^67,71–73^. Based on these observations, we suggest that the joint effect of temperature and especially photoperiod triggered earlier hatching in time-constrained eggs. In addition, adaptive maternal effects might also have played a role in determining egg traits, as this initial developmental stage is more likely to be influenced by maternal contributions compared to later ontogenetic stages—larval and adult—as shown in previous studies^74,75^.

Predator cues prolonged egg development time, especially in late eggs, hence predator cues negated and even reversed the response to time constraints. Changes in egg traits driven by predator cues were previously reported across different taxa, e.g., amphibians^76,77^, salamanders^78^, molluscs^79^ and invertebrates^80^, including *I. elegans*^5,33,36,55^. Yet, the finding that the predator cues can further delay hatching when combined with time stress is, to our knowledge, novel. A possible explanation lies at the physiological level^81^. A slower development in response to predation pressure could result from physiological stress in the eggs as these are potential prey. This stress might prompt a reallocation of energy towards costly defence mechanisms to maintain homeostasis, such as increased production of stress protein to maintain homeostasis^82,83^, rather than a rapid development rate and reproduction^84,85^. Especially delayed egg development under predation pressure in late individuals may be attributed to increased predation risk late in the growth season. Ectothermic predators, such as fish, tend to exhibit increased energy requirements in terms of prey consumption as the season advances in order to accumulate and save energy for wintering and reproduction during the following season^86,87^. Nonetheless, delayed hatching under predation pressure in time-stressed individuals might be adaptive when the fitness cost of predator-induced delayed hatching is lower than the cost of individuals being consumed by predators at hatching.

Surprisingly, damselfly eggs exhibited a more prolonged development under the cues of the signal crayfish than under perch cues, a pattern particularly pronounced in late individuals reared under ambient temperatures. The observation that crayfish cues induced the strongest egg response is in line with our idea that this developmental stage is more susceptible to predation by crayfish than by fish. Omnivorous crayfish commonly consume substrates where immobile damselfly eggs could be laid, moreover they also consume non-active prey, whereas perch primarily prey on mobile targets. Consequently, and consistent with the predator sensitivity hypothesis^23^, the magnitude of the prey response is expected to depend on whether the predator signals come from egg or larval predators^18^. Adaptive prey responses to their stage-specific predators were shown in a green frog *Rana clamitans*: egg predators triggered embryos to hatch at a smaller size and an earlier developmental stage compared to the controls, while larval predators caused a delay in hatching time, leading to larger size and a more advanced developmental stage at hatching^88^. Nevertheless, in our results the direction of the response is unexpected as, if anything, a more prolonged development of eggs in response to crayfish cues, would expose eggs for longer to the risk of predation by crayfish. Such apparently maladaptive responses to predation risk that would increase the exposure duration have, however, been documented (see overview in^89,90^). One reason for this seemingly maladaptive response is that the predator cues caused an upregulation of costly stress proteins to maintain homeostasis, causing the eggs to develop slower. It is worthwhile to note that the here studied crayfish is an alien invasive species. We propose that the response to the invasive crayfish cues is influenced by historical experience with cues from the native Danube crayfish, *Astacus leptodactylus*, which has been observed in the study pond until 2015 (Bonk M., pers. comm.). This is plausible given that the chemical signals released by the two closely related crayfish species^91^ are similar in content^92^. Indeed, our previous studies on geographically nearby populations of *I. elegans* showed that eggs respond similarly to cues produced by two different species of crayfish^55^. Finally, the recognition of cues from predators not present in the study pond may also be attributed to the strong between-population gene flow in *I. elegans*^93^.

Warming reduced the impact of predator cues on egg development time, likely because of accelerated development, leading to a shorter exposure time to the predator cues under elevated temperature. Additionally, the faster biodegradation of predator cues at higher temperatures, as noted in previous case studies^94^, may have contributed to this reduction in the predator effect.

### Immediate and carry-over effects from egg and larval stage up to adult emergence

Under TC, animals are expected to accelerate development and growth rate in the larval stage, which may come at the cost of an increased mortality due to less allocation of energy to somatic maintenance and repair^7,11,95^. Our results confirmed the accelerated development in time-stressed individuals (discussed below), and an associated increase in mortality, but only at the ambient temperature. This implies that mild warming seems to benefit late hatchers in terms of survival. A possible explanation might be that the experimental warming temperature (maximum and average temperatures over the growth season: 26.2 °C and 20.7 °C, respectively) did not deviate considerably from the real temperature recorded at the sampled pond (maximum and average temperature over the growth season 26.4 °C and 19.6 °C, respectively) and could lie within a range of optimal temperatures for survival. This was especially true during the first growth season, i.e., the pre-winter period (Supplementary Fig. S2, Supplementary Table S1), when the larval food acquisition rate is likely the highest^96^. In line with this explanation, earlier studies on *I. elegans* suggest that the upper thermal thresholds for larval survival and growth are slightly below the maximal temperatures observed in both the experimental and field conditions^97^. Due to its characteristics, Płaszowski pond represents a relatively warm freshwater habitat, and *I. elegans* from this site show shorter development times across temperatures when compared to individuals from a nearby colder site, i.e., cogradient variation in the trait^5^. Note that the low survival rate for the early control group reared under the ambient temperature should be interpreted with caution, as several individuals in this treatment group were accidentally lost during the course of the experiment, leading to a relatively low sample size and hence a large error within this group.

Total development time was reduced for the late group, and more so under warming. Along with theory^98^ and similar experimental results^6,99,100^, this outcome is likely adaptive because more time-stressed individuals face a shorter time available before wintering and post-winter development up to emergence. Alternatively, late hatchlings could take another growth season for development and postpone emergence^46,47^, but this did not happen in our results. However, when development time was expressed in DDs, it appeared that late hatchers required fewer DDs than early ones, especially under warming, whereas early individuals under warming needed the most DDs. This further supports the scenario of compensation for time constraint cued by photoperiod in combination with immediate temperature. We also note that a mere four-week-long difference in egg-laying date generated the compensation mechanism in a species that experiences relatively long growth and flying season, which lasts approximately 28 and 18 weeks, respectively^101^ (Supplementary Fig. S1, Supplementary Table S1). In a study by Tüzün et al.^39^ on *I. elegans* from Belgium, larvae from field-collected mothers during the second half of July responded to TC induced by a 6-week-long delayed photoperiod in a qualitatively similar way as late hatchers in the current study, indicating the importance of photoperiod.

The shorter development times in late individuals did not fully compensate for TC in terms of emergence date. On average, later hatchers delayed emergence by 10 days compared to early individuals (while the late clutches were collected ca. 30 days later), with late individuals weighing 12% less than the early ones. The absence of full compensation in development time and a lower mass were reported earlier in other ectotherms (reviewed in Dmitriew 2011), including other damselfly species^102,103^. Such results might be linked to environmental factors such as suboptimal quality and/or quantity of food provided^26,104,105^, genetic constraints in terms of genetic correlations between life history traits^106^ and maternal effects (discussed above). Interestingly, similar differences between early and late season damselflies were found in the size of the field-collected mothers that laid the early and late clutches (current results) and in several other, field-sampled odonate species^107,108^. Our present and previous results show a trade-off between age and mass at emergence^98^ which might eventually lead to an adult fitness cost in term of lower mating success^28,109,110^, but see^111^.

Negative effects of exposure to predator cues in the egg stage were apparent on larval survival until day 14 and less so for survival until emergence, indicating a gradually decreasing but still present carry-over effect from the egg stage. While very few studies demonstrated that predation risk can kill prey in the larval stage^31,112^, this has never been shown for carry-over effects of predation risk imposed in the egg stage. The carry-over effect of predator cues in the egg stage until emergence remained only in late individuals reared under warming and treated with signal crayfish cues. This confirms that crayfish not only delayed hatching but also led to increased mortality under warming through a carry-over effect. This adds to the negative effect of this invasive crayfish on native macroinvertebrate abundance and reduced species number in natural freshwater ecosystems^51^. It is worth to mention that this invasive crayfish shows faster growth rate, higher fecundity, phenotypic plasticity and greater aggressiveness towards their prey compared to native crayfish species^50,51^. This further suggests that this generalist predator is particularly dangerous to native freshwater invertebrates (including *I. elegans* eggs and larvae). However, since signal crayfish primarily invade the northern coastal regions of Poland^50^, the studied damselfly population is not yet exposed to predation stress from this predator. Weaker effects of predator cues through time in damselflies exposed all the time to predator cues probably reflected acclimation to biotic stressors. Such acclimation in predator-treated groups was reported in other organisms, e.g., in fish^113^ and insects^114^, and confirms previous finding in *I. elegans*^5,36^.

Divergent predator cue effects during the egg and larval stages revealed that the variation in development time under predation pressure primarily stemmed from the egg, and not larval reactions to predator cues. The fact that the larval stage was less sensitive in this trait to predator stress could be explained by an acclimation effect (discussed above). Nonetheless, when the total development time was considered (combined egg and larval phases), the carry-over effects of predator cues from the egg up to emergence remained significant. Interestingly, previous studies on *I. elegans* showed that predator cues prolonged larval development time and/or led to lower mass under warming conditions, but this occurred when individuals were reared in groups and were therefore exposed to other stressors, cannibalistic pressure and alarm cue from conspecifics^9,38^, which was absent in the present experiment.

Warming is expected to reduce mass at emergence, the so-called temperature-size rule^115^. Instead, warming did not affect mass at emergence in late damselflies, and early individuals became even heavier at the warmer temperature. This might be because their growth trajectories changed under different temperatures. In this scenario, the difference could indicate the up-regulation of gene expression governing development in the early damselflies experiencing mild warming. This pattern of up-regulated genes categorized into different developmental processes such as anatomical structure development in individuals raised under elevated temperatures has been observed in larvae of *I. elegans* from several populations in southern Poland^38^. The fact that time-stressed individuals did not differ in mass across temperatures indicates a fixed response to TC at the phenotypic and gene expression levels. Such a fixed response might constrain the evolution of this key fitness trait in populations facing rapid environmental change^116,117^.

Life history responses to TC could at least be partially explained by the adjustment of larval behaviour. Higher boldness and activity rates in time-stressed individuals likely increased prey acquisition and, hence, development rates, as earlier reported in other ectotherms, including odonates^13,39,118,119^. However, elevated ecologically risky behaviours, such as increased activity to locate food resources^10,120^, could lead to negative consequences such as physiological costs in terms of a lower investment in immune function^121^ and elevated mortality^11^. In addition, an increased digestive physiology may have contributed to the accelerated life history under TC^122^.

Warming negatively affected boldness in both early and late larvae. In general, the rise in temperature increases metabolic rate exponentially in ectotherms, which often influences behaviour^123,124^. Earlier studies on larvae of *I. elegans* indicated that warming temperature used in our experiment during the behaviour trial (28 °C) was still below the upper thermal thresholds for growth and survival^125^. Here, we obtained a result that remains in contradiction with other studies on *I. elegans* where increased temperature positively affected this behavioural trait^120,126^. However, Debecker and Stoks^120^ and Tüzün and Stoks^126^ conducted their studies under constant thermo-photoperiods, which may yield different results from rearing conducted in a thermo-photoperiod that follows the natural progression of temperature and photoperiod, as demonstrated in the damselfly *Lestes sponsa*^13^*.* Another difference is the more varied diet of the larvae, which from the pre-final instar prior to emergence received additional supplementation with chironomid larvae. This could have led to better conditions of the individuals in our study, and consequently a lower food requirement even under conditions of elevated temperature and increased metabolism. Results similar to ours, with decreased boldness with rising temperatures, have been shown in the invasive crayfish *Procambarus clarkii*^127^.

We expected to see reduced activity in response to predation risk, as earlier indicated in other studies^128,129^, including the study species^130^. However, we did not find such a response. We suggest that this happened because of acclimation in groups that experienced predation risk during both the egg and larval stages (discussed above). Individuals exposed to predator cues during the egg stage only may have retained this acclimation until the short trial period in the larval stage.

## Conclusions

While time constraints imposed by seasonality are widespread, organisms also increasingly face other stressors that may interfere with responses to TC. We here show that both predation risk and warming, to a large extent, can predictably interfere with the responses to TC. Our results indicate that late-hatching individuals compensate by accelerating development, which is facilitated under warming but counteracted under predation risk, especially when imposed by the invasive alien crayfish predator. Notably, we also detected negative carry-over effects of exposure to predation risk in the egg stage into the larval stage. Our results are relevant for global change biology as both warming and invasions by alien predators are key threats in a changing planet, and highlight the importance of evaluating the impact of combinations of stressors across different ontogenetic stages.

## Supplementary Information

Supplementary Information.

## Data Availability

Data are available from the corresponding authors upon request.
