# Supplementary material for: Insect responses to seasonal time constraints under global change are facilitated by warming and counteracted by invasive alien predators
Source: Sci Rep. 2024 Oct 19;14:24565. doi: 10.1038/s41598-024-76057-x (PMC11490650; doi:10.1038/s41598-024-76057-x)
Supplement: Supplementary file 1 — Supplementary Information. [file 41598_2024_76057_MOESM1_ESM.docx]

Supplementary Information

Insect responses to seasonal time constraints under global change are facilitated by warming and counteracted by invasive alien predators

Sniegula S, Stoks R, Golab MJ

Supplementary Methods

*Collection and housing of the predators*

Perch and crayfish were collected a couple of weeks before we started the experiment. Perch was collected from Dobczyce Lake in southern Poland (49°52′18.316′′N, 20°2′30.937′′E) and signal crayfish from Hańcza Lake in northern Poland (54°15′9.522′′N, 22°48′36.86′′E). Animals were transported to the INC PAS by car in travel containers. In the laboratory, the predators were separated by species and kept in aquaria with 52 L of dechlorinated and aerated tap water in the same cabinet at a constant temperature of 20°C. The densities of predators in aquaria were based on the basal metabolic rate equations obtained for perch ^57^ and crayfish ^58^. To keep total metabolic rates balanced, the biomass of crayfish in the aquaria was set two times higher than the biomass of fish. After weighing, we kept two specimens of signal crayfish (wet mass 100 g) and two specimens of perch (wet mass 41.5 g) per experimental aquarium. Perch and crayfish were fed with unfrozen chironomid larvae every second day and live earthworms once a week. We changed 10 L of water in the predator aquaria once a week. Perch were collected and housed with permission from the Local Ethical Committee (ref. 394/2020). Signal crayfish were collected with permission from the Regional Directorate for Environmental Protection in Białystok (ref. WPN.6205.21.2020.ML) and Nature Reserve Hańcza Lake and housed with permission from the Regional Directorate for Environmental Protection in Kraków (ref. OP-I.672.8.2020.MK1). The collection and housing procedures were performed in accordance with relevant guidelines and regulations.


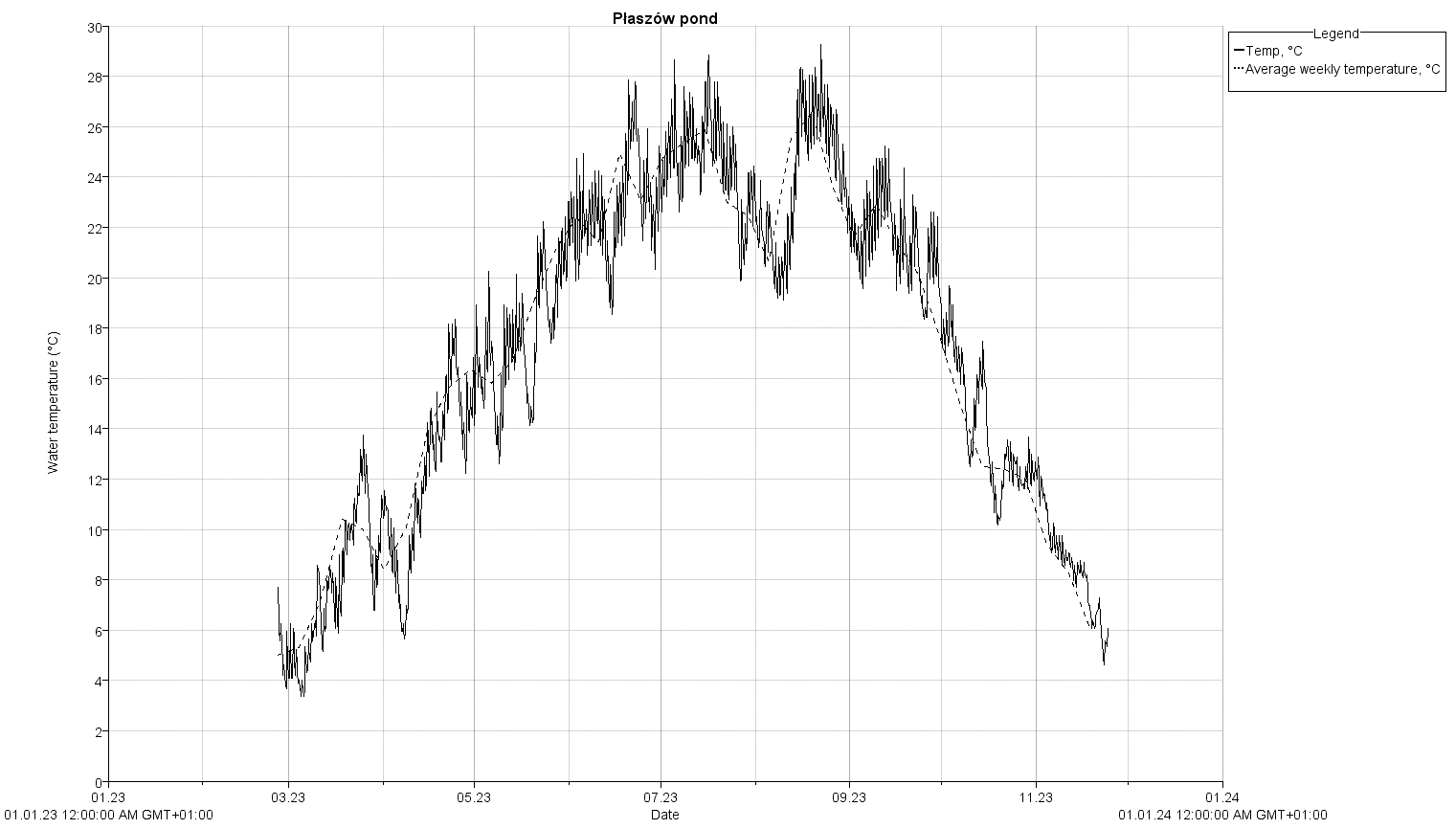


Fig. S1 Water temperature records from Płaszowski pond. The thermo-logger was positioned in the north-west part of the pond, where adult females of *I. elegans* were sampled for egg laying and head width measurements. Installed approximately 40 cm below the water surface, the thermo-logger recorded water temperature four times daily at six-hour intervals (02:00, 08:00, 14:00, and 20:00 local time) from February 25, 2023, to November 24, 2023. The broken line along temperature records illustrates average weekly temperatures. Green and red lines highlight the respective dates when early (less time constraint) and late (more time constraint) individuals were collected in 2020. Horizontal broken line indicates an average threshold temperature at and above which damselflies’ growth happen (Suhling *et al.*, 2015).

Fig. S2 Mean weekly ambient and warming temperatures from Flake modelling (Lake Model Flake, 2009) and field recorded water temperatures in Płaszowski pond in 2023. For detailed description of Płaszowski pond temperature records see caption of Fig. S1.

Fig. S3 Estimated mean female and male larval development time in days (a) and degree-days (b). Error bars show 95%.

Fig. S4 Estimated mean total development time (combined egg and larval) in days (a) and degree-days (b) between different collection dates, temperatures and predator cues. Error bars show 95%. Legend: CC – control egg-control larva, PC – perch egg-control larva, PP – perch egg-perch larva, SC – signal crayfish egg-control larva, SS – signal crayfish egg-signal crayfish larva.

Fig. S5 Estimated mean female and male total development time in days (a) and degree-days (b). Error bars show 95%.

Fig. S6 Estimated mean female and male mass at emergence. Error bars show 95%.

Table S1 Experimental weeks and dates during which photoperiod (light and dark hours), modelled temperature (ambient and warming) and field measured temperature (thermo-logger reads; see Fig. S1 for detailed plot) values were taken. During the experiment, the overwintering period was artificially shortened in comparison to natural overwintering. As a result, during the post-winter treatment starting from week 30, the recorded dates for experimental values did not align with the values observed in nature at the corresponding time points.

| **Week, experimental** | **Date,**  **nature** | **Date, experimental** | **Light  in hours** | **Dark  in hours** | **Temperature °C, ambient, modelled** | **Temperature °C, warming, modelled** | **Temperature °C,  field measured (Płaszowski pond)** |
| --- | --- | --- | --- | --- | --- | --- | --- |
| 0 | 3 July | 3 July | 17:44 | 06:16 | 21.1 | 25.1 | 25.04 |
| 1 | 10 July | 10 July | 17:33 | 06:27 | 21.3 | 25.3 | 25.66 |
| 2 | 17 July | 17 July | 17:17 | 06:43 | 22.2 | 26.2 | 25.26 |
| 3 | 24 July | 24 July | 16:58 | 07:02 | 22.1 | 26.1 | 22.78 |
| 4 | 31 July | 31 July | 16:35 | 07:25 | 22.1 | 26.1 | 22.04 |
| 5 | 7 August | 7 August | 16:12 | 07:48 | 21.2 | 25.2 | 20.90 |
| 6 | 14 August | 14 August | 15:46 | 08:14 | 21.6 | 25.6 | 26.38 |
| 7 | 21 August | 21 August | 15:21 | 08:39 | 20.3 | 24.3 | 26.20 |
| 8 | 28 August | 28 August | 14:53 | 09:07 | 18.4 | 22.4 | 22.74 |
| 9 | 4 September | 4 September | 14:27 | 09:33 | 17.0 | 21.0 | 22.06 |
| 10 | 11 September | 11 September | 14:00 | 10:00 | 15.3 | 19.3 | 22.46 |
| 11 | 18 September | 18 September | 13:33 | 10:27 | 14.1 | 18.1 | 20.95 |
| 12 | 25 September | 25 September | 13:06 | 10:54 | 13.5 | 17.5 | 19.81 |
| 13 | 2 October | 2 October | 12:41 | 11:19 | 12.4 | 16.4 | 17.18 |
| 14 | 9 October | 9 October | 12:15 | 11:45 | 10.4 | 14.4 | 14.77 |
| 15 | 16 October | 16 October | 11:50 | 12:10 | 10 | 14 | 11.86 |
| 16 | 23 October | 23 October | 10:02 | 13:58 | 10 | 14 | 12.25 |
| 17 | 30 October | 30 October | 10:02 | 13:58 | 6.9 | 10.9 | 11.48 |
| 18 | 6 November | 6 November | 00:00 | 24:00 | 6 | 10 | 9.12 |
| 30 | 15 January | 2 April | 12:00 | 12:00 | 10 | 14 | 8.08 |
| 31 | 22 January | 9 April | 13:00 | 11:00 | 10 | 14 | 10.57 |
| 32 | 29 January | 16 April | 13:59 | 10:01 | 10 | 14 | 14.15 |
| 33 | 5 February | 23 April | 15:20 | 08:40 | 14 | 18 | 15.42 |
| 34 | 12 February | 30 April | 15:47 | 08:13 | 16.4 | 20.4 | 16.45 |
| 35 | 19 February | 7 May | 16:12 | 07:48 | 17.6 | 21.6 | 15.86 |
| 36 | 26 February | 14 May | 16:37 | 07:23 | 17.9 | 21.9 | 16.60 |
| 37 | 5 March | 21 May | 16:59 | 07:01 | 18.2 | 22.2 | 19.31 |
| 38 | 12 March | 28 May | 17:18 | 06:42 | 19.4 | 23.4 | 21.29 |
| 39 | 19 March | 4 June | 17:34 | 06:26 | 19.7 | 23.7 | 22.42 |
| 40 | 26 March | 11 June | 17:45 | 06:15 | 20.5 | 24.5 | 21.36 |
| 41 | 2 April | 18 June | 17:51 | 06:09 | 21.0 | 25.0 | 24.89 |
| 42 | 9 April | 25 June | 17:52 | 06:08 | 20.8 | 24.8 | 23.18 |
| 43 | 16 April | 2 July | 17:47 | 06:13 | 21.3 | 25.3 | 25.04 |
| 44 | 23 April | 9 July | 17:37 | 06:23 | 21.1 | 25.1 | 25.66 |
| 45 | 30 April | 16 July | 17:22 | 06:38 | 21.3 | 25.3 | 25.26 |

Table S2 Individuals accidentally lost during the experiment. Legend: CC – control egg-control larva, PC – perch egg-control larva, PP – perch egg-perch larva, SC – signal crayfish egg-control larva, SS – signal crayfish egg-signal crayfish larva. [file: osobniki_eks_gl_nasmiertelnosc_Sz.xls.xlsx]

| Predator group | Early collection | | Late collection | |
| --- | --- | --- | --- | --- |
|  | Ambient  temp. | Warming  temp. | Ambient  temp. | Warming  temp. |
| CC | 2 | 6 | 1 | 0 |
| PC | 0 | 3 | 1 | 1 |
| PP | 1 | 0 | 3 | 0 |
| SC | 1 | 0 | 1 | 2 |
| SS | 0 | 0 | 1 | 1 |

Table S3 Final sample sizes up to the particle measurement phase across the experimental groups and considering individual accidentally lost (see Table S2). Note that for the behavioural traits (Boldness and No of moves), one experimental treatment group (control-control, CC) was not considered in the analysis because this group was represented by zero individuals. Legend: C – control egg, P – perch egg, S – signal crayfish egg, CC – control egg-control larva, PC – perch egg-control larva, PP – perch egg-perch larva, SC – signal crayfish egg-control larva, SS – signal crayfish egg-signal crayfish larva.

|  | Early collection | | Late collection | |
| --- | --- | --- | --- | --- |
|  | Ambient | Warming | Ambient | Warming |
| Survival after 14 days  CC  PC  PP  SC  SS | 21/30  20/30  19/30  20/30  26/30 | 20/30  8/30  8/30  12/30  15/30 | 34/40  22/40  31/40  32/40  35/40 | 37/40  32/40  34/40  37/40  34/40 |
| Survival one day after emergence  CC  PC  PP  SC  SS | 1/28  11/30  10/29  11/29  14/30 | 6/24  3/27  4/30  7/30  7/30 | 5/39  4/39  7/37  3/39  4/39 | 20/40  17/39  23/40  9/38  10/39 |
| Egg development time (in days and degree days)  C  P  S | 21  39  46 | 21  14  28 | 35  52  67 | 37  66  79 |
| Larval development time (in days and degree days)  CC  PC  PP  SC  SS | 1  11  10  13  15 | 6  3  4  7  8 | 5  5  8  4  4 | 19  19  26  12  11 |
| Mass at emergence  CC  PC  PP  SC  SS | 1  11  10  10  13 | 6  3  4  7  8 | 5  3  7  3  4 | 19  16  23  9  10 |
| Boldness  CC  PC  PP  SC  SS | 0  11  10  11  15 | 5  3  3  3  3 | 5  3  5  4  3 | 17  15  14  10  7 |
| No of moves  CC  PC  PP  SC  SS | 0  11  10  11  15 | 5  3  3  4  3 | 5  3  5  4  3 | 17  15  14  10  7 |
